# Understanding malaria treatment-seeking preferences within the public sector amongst mobile/migrant workers in a malaria elimination scenario: a mixed-methods study

**DOI:** 10.1186/s12936-017-2113-4

**Published:** 2017-11-13

**Authors:** Aung Ye Naung Win, Thae Maung Maung, Khin Thet Wai, Tin Oo, Aung Thi, Rungrawee Tipmontree, Ngamphol Soonthornworasiri, Mondha Kengganpanich, Jaranit Kaewkungwal

**Affiliations:** 1 Department of Tropical Hygiene, Faculty of Tropical Medicine, Mahidol University, Bangkok, Thailand; 2 Epidemiology Research Division, Department of Medical Research, No. 5 Ziwaka Road, Yangon, Myanmar; 3 Medical Statistics Division, Department of Medical Research, No. 5 Ziwaka Road, Yangon, Myanmar; 4 Department of Medical Research, No. 5 Ziwaka Road, Yangon, Myanmar; 5 National Malaria Control Programme, Department of Public Health, NayPyiTaw, Myanmar; 6 Bureau of Vector Borne Diseases, Department of Disease Control, Ministry of Public Health, Bangkok, Thailand; 7 Department of Health Education and Behavior Sciences, Faculty of Public Health, Mahidol University, Bangkok, Thailand

**Keywords:** Malaria elimination, Migrant workers, Mixed-methods design, Voluntary health workers, Rural health centers, Public sector, Myanmar, GMS

## Abstract

**Background:**

Migration flows and the emerging resistance to artemisinin-based combination therapy in the Greater Mekong Sub-region (GMS) create programmatic challenges to meeting the AD 2030 malaria elimination target in Myanmar. The National Malaria Control Programme (NMCP) targeted migrant workers based mainly on the stability of their worksites (categories 1: permanent work-setting; categories 2 and 3: less stable work-settings). This study aims to assess the migration patterns, malaria treatment-seeking preferences, and challenges encountered by mobile/migrant workers at remote sites in a malaria-elimination setting.

**Methods:**

A mixed-methods explanatory sequential study retrospectively analysed the secondary data acquired through migrant mapping surveys (2013–2015) in six endemic regions (n = 9603). A multivariate logistic regression model was used to ascertain the contributing factors. A qualitative strand (2016–2017) was added by conducting five focus-group discussions (n = 50) and five in-depth interviews with migrant workers from less stable worksites in Shwegyin Township, Bago Region. The contiguous approach was used to integrate quantitative and qualitative findings.

**Results:**

Among others, migrant workers from Bago Region were significantly more likely to report the duration of stay ≥ 12 months (63% vs. 49%) and high seasonal mobility (40% vs. 35%). Particularly in less stable settings, a very low proportion of migrant workers (17%) preferred to seek malaria treatment from the public sector and was significantly influenced by the worksite stability (adjusted OR = 1.4 and 2.3, respectively for categories 2 and 1); longer duration of stay (adjusted OR = 3.5); and adjusted OR < 2 for received malaria messages, knowledge of malaria symptoms and awareness of means of malaria diagnosis. Qualitative data further elucidated their preference for the informal healthcare sector, due to convenience, trust and good relations, and put migrant workers at risk of substandard care. Moreover, the availability of cheap anti-malarial in unregistered small groceries encouraged self-medication. Infrequent or no contact with rural health centres and voluntary health workers worsened the situation.

**Conclusions:**

Mitigating key drivers that favour poor utilization of public-sector services among highly mobile migrant workers in less stable work-settings should be given priority in a malaria-elimination setting. These issues are challenging for the NMCP in Myanmar and might be generalized to other countries in the GMS to achieve malaria-elimination goals. Further innovative out-reach programmes designed and implemented specific to the nature of mobile/migrant workers is crucial.

**Electronic supplementary material:**

The online version of this article (10.1186/s12936-017-2113-4) contains supplementary material, which is available to authorized users.

## Background

Migrant workers form significant clusters in the economic transition of the Greater Mekong Sub-region (GMS), including Myanmar and five other countries: Cambodia, the Lao People’s Democratic Republic, Thailand, Vietnam, and Yunnan Province of the People’s Republic of China. As a result, the migrant populations in the GMS are increasing and are forecast to reach about five million in 2018 [1]. The National Malaria Control Programmes of Cambodia, Lao PDR, Vietnam, and Myanmar have tailored approaches for mobile and migrant populations (MMPs), due to their higher risk of malaria infection. These programmes promote malaria elimination strategies that worked towards shrinking parasite reservoirs and interrupting transmission taking into account of equity issues [2, 3].

Although artemisinin-based combination therapy (ACT) plays a vital role in reducing the global malaria burden, resistance is emerging in the GMS, including Myanmar, and hampering achievement of the malaria-elimination goal by 2030 [4–7]. MMPs create epidemiological and operational challenges to meet malaria elimination targets in remote areas. Their movements contribute to the spread of the drug-resistance problem from endemic areas to malaria-free regions in the GMS [8–10]. Depending upon their nature of work and seasonal migration, they have difficulties staying in one zone for extended periods. Due to the seasonal nature of their work, migrants may move frequently to seek better income opportunities to sustain their livelihoods. Studies from Vietnam, Cambodia, and Lao PDR have supported evidence of migration flows and the emergence of drug-resistant falciparum malaria [11–14].

The National Malaria Control Programme (NMCP) in Myanmar, defined and classified MMPs into three categories by workplace stability: categories 1 (permanent and more stable worksites with high social capital, where sustainable results can be achieved by malaria control); Category 2: semi-permanent work settings with moderate social capital, where sustainable community-based results can be achieved for malaria control; and category 3 (small, often temporary work sites, less stable worksites with low social capital, where sustainable results for malaria control might not easily be achieved) [15, 16]. Earlier studies in Myanmar had already pointed out that migrant workers, particularly those in category 3, in remote areas, were hard to reach and contributed towards factors that delayed diagnosis within 24 h of onset of fever, to confirm malaria and receive treatment [17, 18]. Despite Myanmar having achieved significant gains in malaria control since 2012 through key interventions for prevention, case management, and surveillance [19], one of the NMCP challenges impeding malaria elimination still hinges on migration issues. The Myanmar Artemisinin-Resistant Containment (MARC) framework (2011–2014) has shifted to Regional Artemisinin-Resistance Initiative (RAI) (2014–2017) supported by the Global Fund. The framework defines MMPs as one important portion of the 2030 malaria-elimination target, and includes the setting up of malaria screening/checkpoints as a key intervention [20–23]. Before MARC activities, and during implementation, two studies from Myanmar discussed the effects of mobility dynamics on socio-behavioural parameters related to malaria and choices of malaria interventions [15, 17]. One qualitative study in Shwegyin Township, Bago Region focused on social determinants of malaria transmission, including treatment-seeking behaviours among gold miners at the pre-elimination stage, in 2013 [24].

To date, no study in Myanmar has addressed access to early malaria diagnosis and treatment (EDAT) services from the public sector among different categories of mobile/migrant workers in remote sites when changing from the MARC to the RAI strategy during the elimination stage. Little has been documented regarding the gaps between the awareness of interventions launched by the NMCP and the uptake of these services by MMPs. There is an urgent need to delineate inequities in access to EDAT amongst MMPs and their poor contact with local health staff from the public sector, which supports scaling up a key intervention for malaria elimination. Therefore, this study aims to assess the migration patterns, malaria treatment-seeking preferences within the public sector and underlying reasons and challenges for EDAT by a diverse group of mobile/migrant workers at remote sites in a malaria-elimination setting in Myanmar.

## Methods

### Study design

The study adopted a mixed-methods explanatory sequential design [25, 26]. A subset of quantitative data (secondary data), acquired through migrant mapping surveys (2013–2015) (n = 9603), was used to ascertain the magnitude of malaria treatment-seeking preferences from the public sector and its contributing factors in the malaria elimination setting. Subsequently, a qualitative strand (2016–2017) was added to enable a more comprehensive understanding of the challenges and underlying reasons in diverse worksites (quanti → Quali).

### Study sites and study population

The study sites during the 2013 and 2014 surveys covered Bago and Tanintharyi Regions, Mon, Kayin and Kayah States. In the 2015 survey, the NMCP included three states/regions—Mon State, Ayeyarwady, and Bago Regions. The study sites for the quantitative strand were selected based on high malaria risk in the malaria elimination stage and undocumented evidence of population movements.

Shwegyin Township, in Bago Region, was selected for qualitative strand as the MMPs engaged in diverse economic activities were estimated to be highest [27]. In the selected study area, the healthcare infrastructure encompasses a 100-bed Township Hospital, a 16-bed Station Hospital, five rural health centres (RHC) and 18 sub-centres. It should be noted that the functions of the RHCs and sub-centres regarding malaria interventions are the same. Of 33 screening/checkpoints for malaria among migrant workers functioning in six states/regions of Myanmar since 2012 [22], two checkpoints for migrant workers in Shwegyin Township were under the jurisdiction of the two RHCs (Than Sate RHC and Done Za Yit RHC). In 2015, the RHC staff trained 72 village health volunteers and supervised for EDAT, malaria prevention and health education activities.

The Vector-borne Disease Control team in Bago Region reported confirmed malaria cases among migrant workers as 5.3 per 1000 tested (98/18,510) in 2016, which was lower than in 2014 and 2015 [23.2 per 1000 tested (510/21,978) and 15.9 per 1000 tested (283/17,752)], respectively [28].

### Sampling strategies, data collection and analysis

For the quantitative strand, the trained interviewers administered pre-tested and modified structured questionnaires to the eligible respondents. Three rounds of migrant mapping surveys employed a multi-stage sampling procedure. First, the NMCP purposively selected townships with significant numbers of migrant workers in six endemic regions. A ratio of 1:2 was considered in selecting migrant clusters classified as categories 1 and 2, versus category 3 [15, 16]. Then, a random sample of migrant households in specific categories was selected in three rounds of surveys, reaching a total of 9603. For the analyses of quantitative secondary data, the variables of interest were extracted from the NMCP database. Cross-tabulations were performed using SPSS version 22.0, to summarize the demographic characteristics, migration patterns, awareness, and treatment-seeking preferences. The Chi square test was used for data analysis, and P ≤ 0.05 was considered significant. Binary logistic regression analysis ascertained significant factors contributing to the deliberate choice of malaria treatment-seeking in the public sector.

**Fig. 1:**
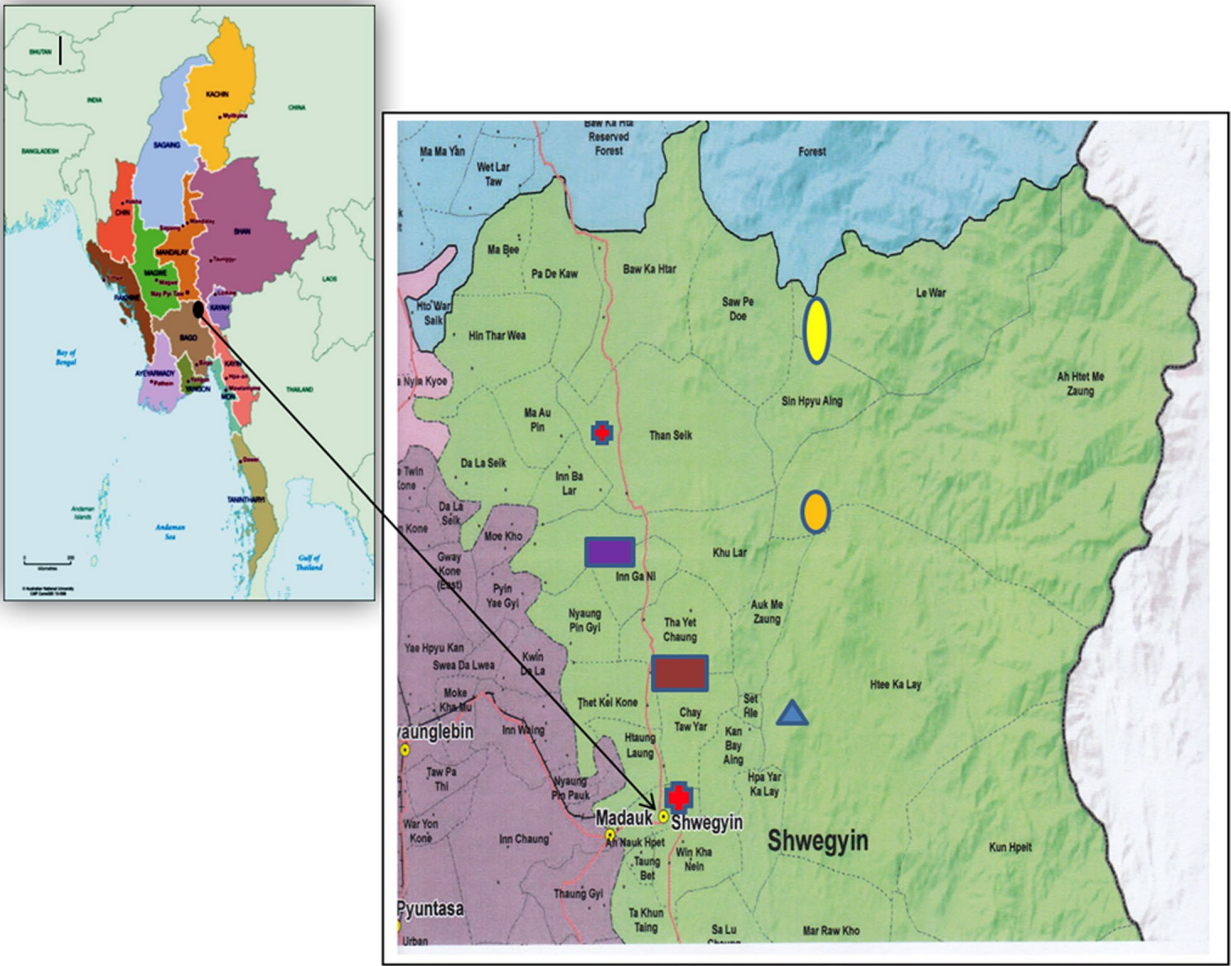
Map showing study sites in Shwegyin Township, Bago Region, Myanmar. http://www.themimu.info/sites/themimu.info/files/documents/Tsp_Map_VL_Shwegyin_Bago_East_MIMU154v04_03May2016_A1.pdf

**Fig. 2:**
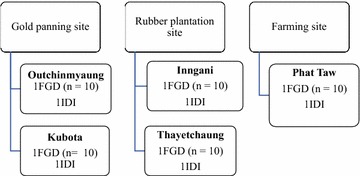
Sampling strategy for qualitative phase of the research, Shwegyin Township. *FGD* focus group discussion, *IDI* in-depth interview

For the qualitative strand, the research team conducted five Focus Group Discussions (FGDs) and five In-Depth Interviews (IDIs) in the Burmese language to underscore and contrast opinions, perceptions, and value systems towards health staff and volunteers, and other unforeseen events. A purposive sampling method was used to recruit participants from categories 2 and 3, who were stratified by nature of worksite: gold panning, rubber plantation, or farming sites (Figs. 1, 2). The selection criteria covered key demographic variables: age, sex, education, and nature of worksite. For FGDs and IDIs, both male and female migrant workers aged between 18 and 60 years, with a previous history of malaria and those who were conversant and able to provide rich information on the health of migrant groups, were recruited. At least three female discussants were included in one FGD of 10 participants to conduct the mixed group. Altogether, 55 migrant workers participated in FGDs and IDIs. Team leaders of migrant groups at gold panning sites, managers of plantation sites, and a migrant worker with a strong history of malaria joined the IDIs. Data were collected from October to November 2016. Informal conversations and observations were supplemented. One moderator and two note-takers were responsible for conducting the FGDs. After a thorough explanation of the purpose of the study, each participant provided written informed consent. The guideline for the IDI was similar to the FGDs, but they were designed for more detailed conversations. Pre-tested guidelines covered migration patterns, common health problems encountered, malaria experiences, their malaria treatment-seeking preferences, and information channels. Privacy, anonymity, and confidentiality issues and concerns were duly observed while conducting the FGDs and IDIs. Translation and back-translation of transcripts were followed by coding and thematic analysis in the Excel spreadsheet. Qualitative data from different sources were integrated for meaningful interpretations.

### Definition of treatment-seeking preferences within the public sector

In this study, treatment-seeking preferences were classified by the operation and management of the healthcare settings. “Public sector” healthcare services in the study sites comprised RHCs, sub-rural health centres, station hospitals, Shwegyin Township Hospital and officially trained voluntary health workers, whereas “private sector” healthcare services in the study sites comprised general practitioner clinics, clinics at worksites, non-government organization (NGO) clinics and malaria volunteers trained by NGOs. Both public and private sector services in this study were collectively referred to as the “formal sector”. “Informal sectors” referred to unlicensed practitioners, drug sellers and itinerant vendors.

### Ethical considerations

The Ethics Review Committee, DMR, Myanmar and the Institutional Review Board of the Faculty of Tropical Medicine, Mahidol University, Thailand approved this study. The NMCP, Myanmar granted permission to use a secondary data subset from migrant mapping surveys (2013–2015).

## Results

Adopting the integration principles and practices in mixed-methods designs [25, 29], quantitative and qualitative findings were reported in two different sections that involved the use of the contiguous adjoining approach.

### Quantitative strand

#### Demographic characteristics and knowledge about malaria amongst migrant workers

**Fig. 3:**
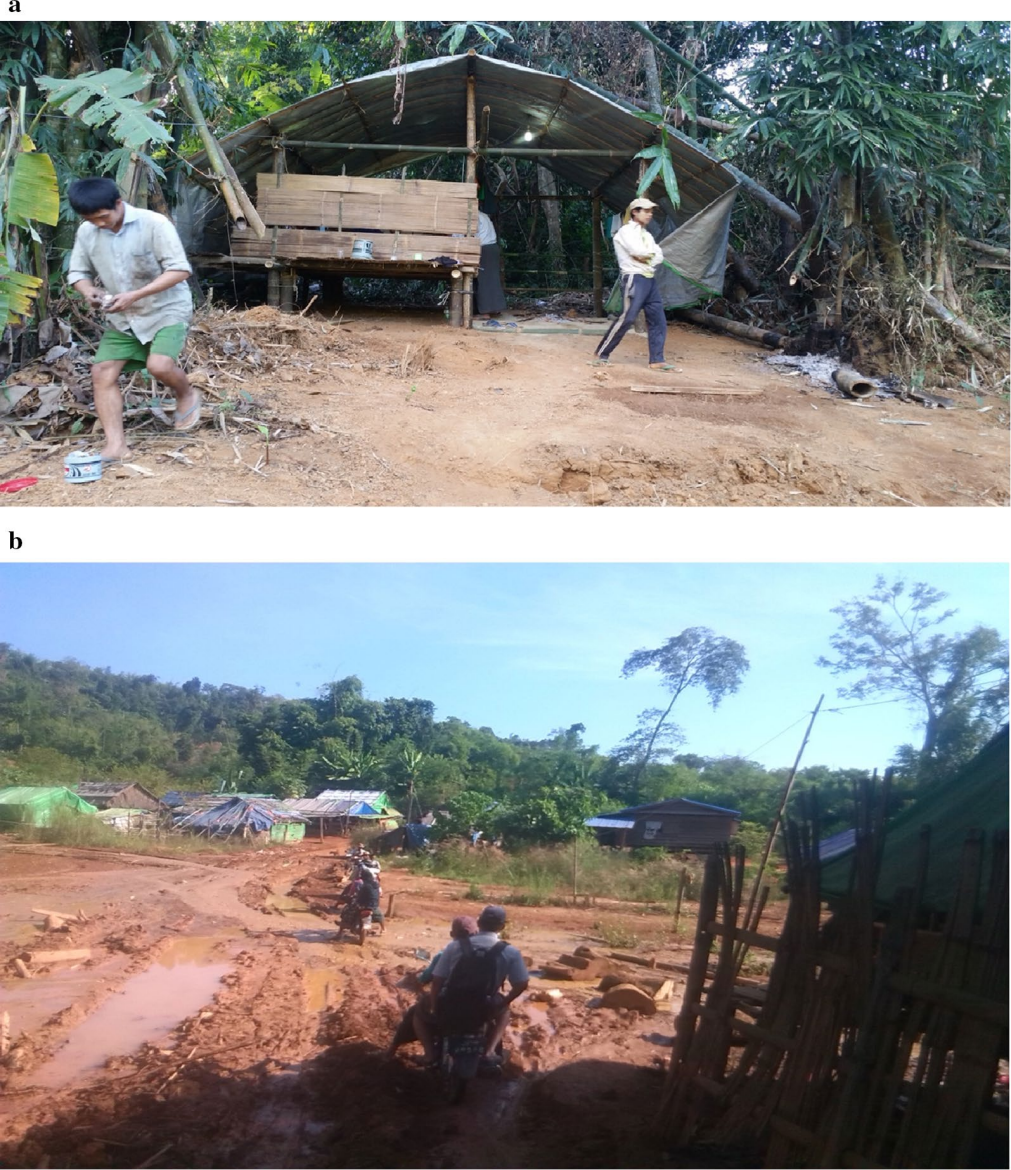
**a**, **b** Living conditions of mobile/migrant workers

Bago Region was more likely to have the occupancy of migrant workers category 3 in the less stable worksites than other regions (2779/3667, 75.8% vs. 3517/5936, 59.2%), as shown in Table 1. The respondents from migrant households in Bago Region (Fig. 3) were significantly more likely to have no or low education than the other regions (1095/3667, 30% vs. 1290/5936, 22%; P < 0.001). They were significantly more likely to report duration of stay ≥ 12 months at the present site (2310/3667, 63% vs. 2886/5936, 49%; P < 0.001) and more seasonal movements than the other regions (1453/3667, 39.6% vs. 2062/5936, 34.7%; P < 0.001). Over half of the migrant workers from Bago and other regions (combined) were aware of health workers’ visits to their worksites (56%; 5403/9603).

**Table 1:** Social demographic characteristics, migration process, correct knowledge and awareness of malaria symptoms and diagnosis for malaria amongst migrant workers with regard to the endemic regions in Myanmar **(**2013–2015)

Characteristic	Total (n = 9603)		Bago Region (n = 3667)		Other regions (n = 5936)		P value
	No.	%	No.	%	No.	%	
Social demographic characteristics							
Category of migrant worksites							
Category 1	590	6.1	183	5.0	407	6.9	
Category 2	2717	28.3	705	19.2	2012	33.9	
Category 3	6296	65.6	2779	75.8	3517	59.2	< 0.001
Number of accompanying family members							
1	778	8.1	189	5.2	589	9.9	
2–3	3497	36.4	1164	31.7	2333	39.3	
4–5	3310	34.5	1417	38.6	1893	31.9	
> 5	2018	21.0	897	24.5	1121	18.9	< 0.001
Education level of respondent							
Illiterate, read and write	2385	24.8	1095	29.9	1290	21.7	
Primary and middle school	6244	65.0	2245	61.2	3999	67.4	
High school and above	974	10.2	327	8.9	647	10.9	< 0.001
Migration patterns							
Duration of stay (months)							
≤ 12	4407	45.9	1357	37.1	3050	51.4	
13–23	1651	17.2	955	26.0	696	11.7	
24–35	1038	10.8	519	14.1	519	8.8	
≥ 36	2309	26.1	836	22.8	1671	28.1	< 0.001
Seasonal migration							
Yes	3515	36.6	1453	39.6	2062	34.7	
No	6088	63.4	2214	60.4	3874	65.3	< 0.001
Intention to move out within 1 year							
Yes	2630	27.4	761	20.8	1869	31.5	
No	6973	72.6	2906	79.2	4067	68.5	< 0.001
Awareness of malaria							
Health staff visited for malaria-related reasons							
Yes	5403	56.3	1655	45.1	3748	63.1	
No	4200	43.7	2012	54.9	2188	36.9	< 0.001
Received any form of IEC on malaria							
Yes	2979	31.0	1230	33.5	1749	29.5	
No	6624	69.0	2437	66.5	4187	70.5	< 0.001
Malaria symptoms known^a^							
Yes	2809	29.3	1225	33.4	1584	26.7	
No	6794	70.7	2442	66.6	4352	73.3	< 0.001
Awareness of means to diagnose malaria^b^							
Yes	6870	71.5	2082	56.8	4788	80.7	
No	2733	28.5	1585	43.2	1148	19.3	< 0.001

Public sector referred to rural health centres, sub-rural health centres, station hospitals, a township hospital and trained voluntary health workers

Private sector referred to general practitioners, clinics at work sites, clinics of non-government organizations (NGO) and malaria volunteers trained by NGOs

*IEC* information, education, communication

^a^Malaria symptoms included combined fever, chills and rigour and headache

^b^Means to diagnose malaria referred to being aware of either the rapid diagnostic test or light microscopy

Although migrant households in Bago Region were significantly less likely to report having contact with health workers for malaria-related reasons than those in other regions (1655/3662, 45% vs. 3748/5936, 63%), they were more likely to receive any form of malaria-related IEC than those in other regions (1230/3662, 34% vs. 1749/5936, 30%; P ≤ 0.001). Approximately 70% of the respondents from Bago and other regions (combined) could not correctly cite three common symptoms indicating malaria (fever, chills and rigor, headache). Compared with other regions, migrant workers from Bago Region were significantly less likely to mention either a rapid diagnostic test (RDT) or microscopy as methods to confirm malaria (2068/3667, 56.8% vs. 4788/5936, 80.7%; P < 0.001).

#### Treatment-seeking preferences

As shown in Table 2, Bago or “other regions” tended to be more likely to seek malaria-related treatment from either the public sector or a combination of public and informal sectors. Regarding treatment-seeking from only the public-health sector among category 3 migrant workers, the proportions were different between 17.5% (16.1–18.9) in Bago Region vs. 13.1% (12.0–14.3) in other regions. Similar situations were observed amongst category 2 migrant workers, with 22.4% (19.4–25.7) in Bago Region vs. 16.7% (14.9–18.2) in other regions, and amongst category 1 workers with 33.9% (27.1–41.2) in Bago vs. 26.3% (22.1–30.9) in other regions. About similar proportions were observed amongst those in all categories who went to both public and informal sectors combined.

**Table 2:** Treatment-seeking preferences of migrants from different sectors in Bago and other regions of Myanmar (2013–2015) by category of migrant worksites

Region	Category of migrant worksites								
Bago Region (N = 3667)	Category 1 (N = 183)			Category 2 (N = 705)			Category 3 (N = 2779)		
Type of treatment-seeking^a,Ψ^	n	%	95% CI	n	%	95% CI	n	%	95% CI
Public sector	62	33.9	27.1–41.2	158	22.4	19.4–25.7	485	17.5	16.1–18.9
Private sector	27	14.8	10.0–20.7	60	8.5	6.6–10.8	302	10.9	9.7–12.1
Informal sector	7	3.8	1.6–7.7	40	5.7	4.1–7.7	144	5.2	4.4–6.1
Public + informal sectors	63	34.4	27.6–41.8	186	26.4	23.2–29.8	579	20.8	19.3–22.4
Private + informal sectors	33	18.0	12.8–24.4	95	13.5	11.0–16.2	421	15.2	13.8–16.5

Region	Category of migrant worksites								
Other regions (N = 5936)	Category 1 (N = 407)			Category 2 (N = 2012)			Category 3 (N = 3517)		
Type of treatment seeking^a,Ψ^	n	%	95% CI	n	%	95% CI	n	%	95% CI
Public sector	107	26.3	22.1–30.9	333	16.7	14.9–18.2	461	13.1	12.0–14.3
Private sector	34	8.4	5.9–11.5	143	7.1	6.0–8.3	184	5.2	4.5–6.0
Informal sector	5	1.2	0.0–2.8	37	1.8	1.3–2.5	23	0.7	0.0–0.1
Public + informal sectors	111	27.3	23.0–31.9	361	17.9	16.3–19.7	470	13.4	12.3–14.5
Private + informal sectors	37	9.1	6.5–12.3	174	8.7	7.5–9.9	203	5.8	5.0–6.6

^Ψ^P < 0.05

^a^Multiple answers

#### Factors contributing to preference of treatment-seeking from public-sector health services

Table 3 presents the results of the binary logistic regression procedure. Compared with other regions, migrant workers from Bago Region were significantly more likely to seek malaria treatment from the public sector after adjusting for other variables [adjusted OR = 1.2 (95% CI 1.1–1.4)]. Compared with category 3, migrant workers from the other two categories were significantly more likely to seek treatment from the public sector: for category 1, adjusted OR = 2.3 (95% CI 1.9–2.7); and for category 2, adjusted OR = 1.4 (95% CI 1.2–1.6). Differences in the educational level of the migrant workers did not have a significant impact on their intended choices to seek treatment from the public sector. Likewise, whether migrant workers moved seasonally or not, had no significant influence on their preference towards the public sector. Long duration of stay (≥ 12 months) at the worksite showed a significantly higher likelihood to seek treatment from the public sector compared with those with duration < 1 year [adjusted OR = 3.5 (95% CI 3.1–4.1)].

**Table 3:** Factors contributing to malaria treatment-seeking from public sector healthcare services by migrant workers in malaria endemic regions of Myanmar (2013–2015)

Characteristics	Sought treatment from public sector				Crude OR (95% CI)	P value	Adjusted OR (95% CI)	P value
	No (n = 7997)		Yes (n = 1606)					
	No.	%	No.	%				
General characteristics								
Region								
Other regions^a^	5035	84.8	901	15.2	1.0		1.0	
Bago Region	2962	80.8	705	19.2	1.3 (1.2–1.5)	< 0.001	1.2 (1.1–1.4)	< 0.001
Category of work site								
Category 3^a^	5350	85.0	946	15.0	1.0		1.0	
Category 2	2226	81.9	491	18.1	1.8 (1.5–2.2)	< 0.001	1.4 (1.2–1.6)	< 0.001
Category 1	421	71.4	169	28.6	2.3 (1.9–2.7)	< 0.001	2.3 (1.9–2.9)	< 0.001
Education level								
Illiterate, read, write^a^	2014	84.4	371	15.6	1.0		1.0	
Primary and middle	5203	83.3	1041	16.7	1.4 (1.1–1.6)		1.0 (0.9–1.2)	1.00
High school and above	780	80.1	194	19.9	1.2 (1.1–1.5)	0.01	1.1 (0.9–1.3)	1.00
Migration patterns								
Seasonal migration								
No^a^	5004	82.2	1084	17.8	1.0		1.0	
Yes	2993	85.1	522	14.9	0.8 (0.7–0.9)	0.00	1.1 (0.9–1.3)	0.08
Duration of stay (year)								
< 1^a^	4053	92.0	354	8.0	1.0		1.0	
≥ 1	3944	75.9	1252	24.1	3.6 (3.2–4.1)	0.00	3.5 (3.1–4.0)	< 0.001
Awareness of malaria								
Received any form of IEC on malaria								
No^a^	5689	85.9	935	14.1	1.0		1.0	
Yes	2308	77.5	671	22.5	1.8 (1.6–2.0)	< 0.001	1.2 (1.0–1.3)	0.02
Health staff visited for malaria related reasons								
No^a^	3617	86.1	583	13.9	1.0		1.0	
Yes	4380	81.1	1023	18.9	1.5 (1.3–1.6)	< 0.001	1.0 (0.9–1.2)	0.65
Malaria symptoms known								
No^a^	5741	84.5	1053	15.5	1.0		1.0	
Yes	2256	80.3	553	19.7	1.3 (1.2–1.5)	< 0.001	1.7 (1.4–2.2)	< 0.001
Aware of diagnosis of malaria								
No^a^	2392	87.5	341	12.5	1.0		1.0	
Yes	5605	81.6	1265	18.4	1.6 (1.4–1.8)	< 0.001	1.4 (1.2–1.7)	< 0.001

*95% CI* 95% confidence interval, *OR* odds ratio

^a^Reference category

Regarding awareness of malaria, those who had received any form of malaria message were significantly more likely to seek treatment from the public sector than those who did not [adjusted OR = 1.2 (95% CI 1.0–1.3)]. However, an awareness of health staff visiting their worksite for malaria-related reasons did not influence migrant workers’ preference to seek treatment for malaria from the public sector. In addition, significant differences in seeking treatment from the public sector were found among migrant workers who could cite malaria symptoms [adjusted OR = 1.7 (95% CI 1.4–2.2)], and those who knew the means of diagnosis for malaria [adjusted OR = 1.4 (95% CI 1.2–1.7)].

### Qualitative strand

Tables 4 and 5 summarize the social and demographic characteristics of focus group discussants and in-depth interviewees in Shwegyin Township. The qualitative findings further revealed challenges to seeking malaria treatment from the public sector, and the underlying reasons.

**Table 4:** Characteristics of focus group participants in Shwegyin Township, Bago Region, 2016 (n = 50)

Characteristic	Frequency	%
Place of origin		
Ayeyarwaddy region	4	8
Bago Region	18	36
Kachin state	7	14
Kayah state	1	2
Magway region	2	4
Mandalay region	4	8
Mon state	12	24
Sagaing region	1	2
Shan state	1	2
Sex		
Male	35	70
Female	15	30
Age group (years)		
< 30	21	42
30–39	11	22
40–49	15	30
≥ 50	3	6
Education		
Illiterate, read and write	8	16
Primary school	29	58
Middle school and above	13	26
Occupation site		
Gold panning site	20	40
Rubber plantation site	20	40
Farming site	10	20

**Table 5:** Characteristics of in-depth interviewees in Shwegyin Township, Bago Region, 2016 (n = 5)

Serial no.	Participant initial	Gender	Age (in years)	Education	Occupation
1.	T	Male	35	Primary	Gold miner
2.	A	Male	23	Primary	Gold miner
3.	Th	Female	37	Primary	Rubber tapper
4.	K	Male	59	Graduate	Manager
5.	M	Male	37	Primary	Farmer

#### Migration patterns

Most of the migrant workers moved into Shwegyin Township from Kachin State in the northernmost part of Myanmar: some from Mon State in the southern part and also from Magway and Mandalay Regions in the dry and arid part of Central Myanmar. During the FGDs, those engaged in goldmine work in the area reported the longest duration of 9 years. Also, rubber plantation workers reported their range of stay in respective sites as 3 months to 10 years. Once the rubber tapping season was over, some moved around and tended to seek work in nearby villages and then returned to their plantation sites. Migrant workers in agricultural fields moved seasonally from village to village within Shwegyin Township. Usually, farm workers preferred to stay in the same area and switched to other kinds of manual labour, such as charcoal making, bamboo cutting, and fishing. Their nature of worksite, worksite relations, favourable living conditions (housing, clean water, and sanitation), and the availability of seasonal earning opportunities, provoked variations in duration of stay, periodic migration patterns, and primarily circular/cyclic movements.

#### Malaria experiences and treatment-seeking in remote worksites

Some migrants had malaria testing experience due to prolonged fever at their former destination, with advice from health staff visiting their worksite. The respondents unanimously accepted that they were prone to malaria due to the nighttime nature of their work, their sleeping patterns without nets due to tiredness from work, and their work environment close to forests, and inadequate daylight. Apparently, familiarity with malaria did not prompt them to confirm suspected fever by RDT, and its availability at RHCs and malaria volunteers. Once they experienced suspected malaria, they preferred to use their mobile phones to request unlicensed practitioners visit their worksites, and they could afford to pay them per visit. Besides, they valued not having to leave their worksite and thereby retaining their daily earnings. All of the respondents said that even though they had received treatment free of charge at the RHC or township hospital, they could not afford the costs of transportation to and from these facilities. A round-trip journey by motorcycle ranged from at least 10–15 US$. Also, the health facilities were quite distant from their worksites, and travel time varied from 2 to 4 h. Moreover, the open hours at the RHCs were unsuitable for them.

*“No, we don’t want to contact a health staff from RHC. We are busy at worksites”.* (FGD, Gold panning site)

*“Charges for motorcycle taxis were expensive to visit RHC. Besides, opening hours were not suitable for us to visit. When our working hours were over, it already closed.”* (IDI, Migrant group leader, Gold panning site)

Second, the migrants first choice was self-medication rather than relying on either the formal public and private sectors, or the informal healthcare sector. Together with mono-therapeutic drugs for malaria, they could purchase the combination of analgesics, antibiotics and antihistamines from small groceries, as prescribed by drug sellers or itinerant vendors. Migrant workers had easy access to cheap anti-malarials without any confirmation of fever.

*“At first, we buy drugs from the shops in the gold mines. If fever is not relieved we go to RHC or the private clinic depending on the severity and budget.”* (FGD, Gold panning site)

Also, they sometimes preferred traditional medicine packages, and relied on the specific brands that had helped them during previous fever episodes. When their choice of treatment worked, they avoided going to the RHC or hospital, whether it was malaria or not. If their fever was not relieved and the symptoms became more severe, they sought treatment from the RHC. Nearly all discussants said they had less frequent or no contact with public-sector health staff or volunteers, and also felt reluctant to communicate with them. The main reasons for reluctance included infrequent visits to worksites and lack of confidence in their treatment. However, qualitative interviews did not reveal any issues, such as language barriers, negative attitudes of health staff, and ineffective or negative interactions. Nevertheless, some did not even know the staff from the RHCs or the volunteers, which may lead to difficulties in formal encounters and consultations.

#### Challenges for early diagnosis and adequate treatment

During FGDs, most of the migrant workers were unable to state the methods by which malaria can be diagnosed. Only a few migrants from gold-panning sites were aware of RDT, but they had never witnessed it being performed. Nevertheless, some discussants from the rubber plantations said they would like to seek malaria diagnosis at the RHC or the malaria volunteers free of charge, and they did not doubt the usefulness of RDT in malaria diagnosis. All FGD participants stated single drugs (monotherapy), such as artesunate, artemether, and mefloquine, due to the easy availability at small unregistered, low-cost groceries, although banned by the government (also confirmed by observations). Only migrant workers who consulted health staff, who underwent RDT testing, and who received treatment, were aware of the ACT and the health staff’s recommendation to complete the full course.

*“We seek for malaria testing at the nearest RHC. However, when malaria volunteers come to the sites, they provide RDT, free of charge. RDT is useful because it can identify the infection due to malaria parasites.”* (FGD, Rubber plantations)

*“I took the ACT which was a yellow card, four tablets each for morning and evening for three consecutive days. Health staff from RHC told me that if I did not complete the full course, malaria wouldn’t be cured”.* (IDI, Malaria positive migrant worker, Rubber plantations)

*“I’ve tried testing with RDT, and the health staff relayed the message as malaria positive, gave drugs and after that fever was relieved.”* (IDI, Malaria-positive migrant worker, Gold panning site)

Only a few respondents mentioned that malaria volunteers visited only once per year and they had difficulty contacting both health staff and malaria volunteers.

#### Unravelling information and communication channels and proposed solutions

Regarding awareness and the alarming danger of malaria, some participants discussed the fact that they had learned about the issue through health staff and volunteers, and also from Myanmar’s television station. Their work sites were too far away from the villages with RHCs or sub-RHCs, and they encountered telecommunications barriers. Amongst the communication channels, most of the migrant workers mentioned that transmitting knowledge from radio programmes was the best way to reach them at remote sites. Some of the respondents had an idea to train the team leader, or one of their co-workers as a malaria volunteer, to learn from the health staff. They have stated all precautionary measures that they practiced and their awareness related to diagnosis and treatment seeking for malaria. The following excerpts underscored ways to improve their knowledge gaps and demand for information:

*“We want to know about how to differentiate fever by confirmation of malaria.”* (FGD, Gold panning site)

*“We want to know about the exact dosage and use of drugs for malaria so that we can buy from drug shops and try correctly.”*

(FGD, Rubber plantations)

*“We want to know about the testing of malaria by training plantation workers as communicators delegated by the health staff so that we can confirm malaria quickly.”* (IDI, Manager of Rubber plantations)

*“We want health talks in here during free time. These talks can be held by negotiation with our manager by staff from rural health centres.”* (FGD, Rubber plantations)

## Discussion

This is the first study reported from Myanmar focusing on the reach and uptake of EDAT interventions provided by fully equipped and well-trained health staff and volunteers from the public sector, amongst the MMPs residing in the planned malaria-elimination zone. Gold mines, rubber plantation sites, forest-related work, and agricultural settings, attracted most of the MMPs in such malaria-endemic regions, among others [23]. In this study, migrant workers who engaged in less stable work sites formed a significant proportion in malaria risk areas and were more likely to come across into the Bago Region than others. Since the Bago Region is a transit area, it acts as a hub for migrant workers from other states/regions of Myanmar, where many migrants come to seek new job opportunities. Besides, it is also easily accessible to other regions [27]. The predominance of category 3 worksites in the Bago Region, which was highly unstable, necessitated the introduction of more effective approaches than at present to improve the coverage of EDAT and to deter local transmission during the pre-elimination stage.

Migrant workers from the Bago Region were less likely to prefer health services from the public sector for malaria treatment than those from other regions. While living in one area for an extended period, there is a potential to increase the frequency of contact with health staff, which could further influence treatment-seeking from non-public sector health services. The most unregulated private informal health sector was also reported as popular among migrant workers in other resource-constrained settings [30]. This mental distance or emotional barrier to the health staff and volunteers from the public sector was also noted in a study from Cambodia [31]. However, due to seasonal variations in migration patterns in malaria-endemic areas, these movements might reduce the opportunities of contacting RHCs or voluntary health workers, which was also noted by other studies in the GMS [13, 30, 32]. The low proportion of migrant workers seeking treatment from the public sector (< 35%) in all endemic regions and Bago Region, in particular, called for a detailed exploration of the key drivers. The main reasons uncovered were the remoteness of their worksites, unaffordable transport charges, unsuitable open hours of the screening clinics, and social distance. Lack of ready cash was a barrier, apart from travel constraints, was also stated in a recent study in rural Cambodia [33]. In addition, there was a higher chance of self-medication for suspected malaria among MMPs rather than opting for formal or informal health care, as revealed during qualitative data collection. The usual reason included the availability and popularity of monotherapeutic drugs in unregistered small grocery shops. Other studies from the GMS revealed similar evidence [12–14]. A study in Africa also reported that self-medication practices in the remote worksites of MMPs inclined to an irrational use of substandard and counterfeit anti-malarials [34].

More importantly, this study elucidated the relevant policy and programme implications for the successful implementation of malaria-elimination strategies in Myanmar. First, characterization of mobile/migrant workers who sought malaria treatment from the public sector covered those from the permanent and more stable work settings (categories 1 and 2). Apparently, the accessibility of unlicensed practitioners, and trust and good relations with them, increased the likelihood of MMPs’ seeking health care from the informal sector. Apart from these issues, their preference for unlicensed practitioners was driven mostly by their value system for convenience regarding no travel restraints and individual cost-savings through the lack of absenteeism from worksites. In this connection, seeking malaria care from the informal sector may decrease exposure to substantial care and ACT according to NMCP guidelines [17]. This phenomenon may accelerate the threat of ACT resistance and its spread, especially among goldmine workers in Shwegyin Township [35].

Second, the interaction between duration of stay > 1 year and seasonal mobility patterns among migrant workers from unstable work settings may decrease their opportunities to contact the formal health sector. According to estimates in 2012, before MARC interventions, 826 migrant clusters were mapped as malaria hotspots in Shwegyin Township, in a populated of around 15,899 migrants [27]. Qualitative data also suggested some of the mobile/migrant workers as multi-task holders and their circular and multidirectional movements, reduced their opportunities to contact health staff and malaria volunteers. Short transitory stays among seasonal migrant workers in Bago Region were noted as one of the challenges of the treated-net distribution programme for MMPs [16]. Similarly, underexposure and low access to malaria interventions was noted among seasonal workers in Pailin Province, Cambodia [5]. The qualitative analysis in this study suggested that the MMPs in this study had quick movement patterns coupled with shorter duration of stay at the particular site; this may risk making them potential transmitters of drug resistance problems from one area to another as reported in other GMS countries [6, 14, 36]. Providing incentives and scheduling malaria volunteers to reach the target groups of migrant workers with high mobility is imperative to improve access to public sector health care. The emphasis of this issue among migrant clusters is critical in achieving the malaria elimination target by 2030 in relation to the problem of drug resistance [37].

Third, a low proportion of awareness of malaria symptoms and means of diagnosis of malaria significantly contributed towards the low use of public sector health services by MMPs especially the screening check-points. However, as revealed during IDIs, a certain proportion was willing to consult health staff for correct diagnosis and the complete course of ACT treatment. The current approach impinged the weak effect of IEC among migrant workers in less stable clusters, which was also noted in a study in Myanmar [15].

Migrant workers required testing with RDT within 24 h of the onset of fever, to rule out malaria or to receive adequate treatment in line with NMCP guidelines in response to malaria diagnosis. Usually, RDTs are used quite often to ascertain the requirement for treatment of suspected malaria at point-of-care [36, 38, 39]. Therefore, awareness-raising activities that are adaptable, financially feasible and acceptable to migrant worksite, require attention to promote their best practices and to increase investment for the implementation of mobile screening programmes by health staff and volunteers in partnership with the formal private sector. Nevertheless, shortcomings were found in the reach of malaria messages and impact, which agreed with A recent study from Cambodia targeting migrant workers [33]. It is apparent that good knowledge of mobility dynamics as well as a well-established malaria-information system, is needed to capture real-time data [2, 30, 40].

The major strength of the quantitative part of this study is the large set of data among MMPs in a national survey in six endemic regions of Myanmar, which provided sufficient information for the generalizability of important contributory factors on treatment-seeking preferences from the public sector. The sequential qualitative part highlighted the considerable misunderstanding and constraints in acquiring EDAT amongst mobile/migrant workers in remote sites. It also revealed potential factors that might hamper malaria elimination. However, the limitation of this study was related to the specific types of worksites being sampled; and thus there might be certain factors amongst MMPS in other settings that may not be observed in this study. Moreover, observations and discussions were made only among migrants but not health staff and village malaria volunteers at the screening checkpoints; this resulted in a lack reflections by healthcare providers regarding the impact of migration on malaria treatment-seeking behaviour.

## Conclusions

There are fluctuations in the at-risk populations within static communities in remote areas, which are of significant concern and problematic for malaria elimination. This study suggested the key drivers that increased the risk of poor utilization of public-sector services for treating malaria included the nature of the work setting, and prolonged duration of stay linked to high seasonal mobility and low awareness. These are serious challenges confronting the NMCP in Myanmar, as the country moves towards malaria elimination. The timely introduction of corrective actions fit into the local setting, giving priority to strengthening malaria volunteers, is crucial to improving the current situation. Periodic assessment of gaps is essential in access to formal health services and quality care for fever that is suspected to be malaria, among mobile migrant workers with a high degree of locational instability. Further design and implementation of innovative outreach programmes, together with awareness-raising activities specifically tailored to mobile/migrant workers, and the evaluation of their impact comparing local communities/static communities at remote sites, is crucial.

## Additional file

**Additional file 1.** FGD and IDI guidelines.
