# Supplementary material for: Understanding malaria treatment-seeking preferences within the public sector amongst mobile/migrant workers in a malaria elimination scenario: a mixed-methods study
Source: Malar J. 2017 Nov 13;16:462. doi: 10.1186/s12936-017-2113-4 (PMC5683526; doi:10.1186/s12936-017-2113-4)
Supplement: Supplementary file 1 — Additional file 1. FGD and IDI guidelines. [file 12936_2017_2113_MOESM1_ESM.zip › GUIDELINES FOR IN DEPTH INTERVIEW.docx]

**GUIDELINES FOR IN DEPTH INTERVIEW**

Background Characteristics

Name, Sex, Age. Highest education attended,

Origin of residence. Nearest health facility, average distance to go,

Time duration to arrive health facility by cycle.

Topic 1 Mobility

1. How long had u been here? Where did u migrate from? How long will u stay here? After this place, where’ll you migrate again? When you go?
2. When you are in here, is there any person going to back origin of residence or migrate to another region? Reasons.
3. Before you are going back to origin of residence or migration, yourself or any of family members has habit of medical checkup. Any difficulties, explain.
4. At your origin of residence or former place of migration, did u make testing for malaria? Please share the experiences.

Topic 2 Common Health Problems

1. Describe the most common health problems encountered in here.
2. When you are staying in here, do you think malaria is a big issue to face?
3. Compare with other health problems and share your experiences and feelings.
4. If your family or team members suffer fever, what will you do?
5. For the treatment seeking, how you discuss and make a decision in your team or family? Who is the most responsible in judging it? Describe the experiences.

Topic 3 Malaria and vulnerability

1. What is malaria? (Signs and symptoms, Severity…)
2. Why does malaria happen?
3. How can malaria transmit from one person to another?
4. How can we prevent from transmission of malaria?
5. Comparing with migrant workers from other areas, how about the probability of having malaria?

Topic 4 Malaria experiences

1. Is there any person who suffering malaria in your family or migrant group?
2. How can you know that it is malaria?
   - - 1. When yourself, family or in this group suffer malaria, where u go for initial treatment seeking and also from who. Is there any desire to contact with malaria volunteers and why?
       2. Is there any person who hospitalized because of malaria in here? Did u hear any deaths of malaria?
       3. How can you diagnose and confirm malaria? (Microscope, RDT.)
       4. How do you know that RDT can detect and diagnose malaria from who and where?
       5. Is there any convenience for the usage of RDT and where can u get the service and cost? Why?
       6. Do you think RDT is reliable? Give reasons for explanations.
       7. Do you hear about using ACT for the treatment of malaria? Where? From Who? For the full course, how should u take. Why?
       8. When you are suffering malaria, can u guess the cost and affordable?
       9. When you go nearest health facility for treatment seeking, any difficulties of travel distance, availability of health officers at the facility, permission to go there by the site owner. How can we solve these barriers?
       10. Can I ask your salary? When treatment seeking, u pay by yourself or site owner share it.
       11. Do you know malaria volunteers? Have you made contact with them? Any difficulties, how can u overcome them?

Topic 5 Information channels and preferences

1. Who give alarm for the awareness of malaria?
2. In your family or migrant groups, any discussions about malaria for the diagnosis, treatment seeking and prevention?
3. Any extra information does u want to know about malaria? (Diagnosis, Treatment)
4. Give me suggestions for making the improvement of knowledge and awareness of malaria depends on occupation, leisure time, media or any methods.

Suggestions

When yourself, family or migrant groups suffer fever, how u want to make the health authorities for the improvement and awareness of diagnosis and treatment seeking of malaria.
